# Prognostic Nomogram for Postoperative Patients With Gastroesophageal Junction Cancer of No Distant Metastasis

**DOI:** 10.3389/fonc.2021.643261

**Published:** 2021-04-16

**Authors:** Qiang Guo, YuanYuan Peng, Heng Yang, JiaLong Guo

**Affiliations:** ^1^ Department of Thoracic Surgery, Taihe Hospital, Hubei University of Medicine, Shiyan, China; ^2^ Department of Gastroenterology, The Affiliated Xinchang Hospital of Wenzhou Medical University, Wenzhou, China

**Keywords:** gastroesophageal junction cancer, nomogram, overall survival, Cox, AUC

## Abstract

**Background:**

Gastroesophageal junction (GEJ) was one of the most common malignant tumors. However, the value of clinicopathological features in predicting the prognosis of postoperative patients with GEJ cancer and without distant metastasis was still unclear.

**Methods:**

The 3425 GEJ patients diagnosed and underwent surgical resection without distant metastasis in the Surveillance, Epidemiology and End Results (SEER) database from 2010 to 2015 were enrolled,and they were randomly divided into training and validation cohorts with 7:3 ratio. Univariate and multivariate Cox regression analysis were used to determine the predictive factors that constituted the nomogram. The predictive accuracy and discriminability of Nomogram were determined by the area under the curve (AUC), C index, and calibration curve, and the influence of various factors on prognosis was explored.

**Results:**

2,400 patients were designed as training cohort and 1025 patients were designed as validation cohort. The percentages of the distribution of demographic and clinicopathological characteristics in the training and validation cohorts tended to be the same. In the training cohort, multivariate Cox regression analysis revealed that the age, tumor grade, T stage and N stage were independent prognostic risk factors for patients with GEJ cancer without distant metastasis. The C index of nomogram model was 0.667. The AUC of the receiver operating characteristic (ROC) analysis for 3- and 5-year overall survival (OS) were 0.704 and 0.71, respectively. The calibration curve of 3- and 5-year OS after operation showed that there was the best consistency between nomogram prediction and actual observation. In the validation cohort, the C index of nomogram model, the AUC of 3- and 5-year OS, and the calibration curve were similar to the training cohort.

**Conclusions:**

Nomogram could evaluate the prognosis of patients with GEJ cancer who underwent surgical resection without distant metastasis.

## Introduction

Gastroesophageal junction (GEJ) carcinoma was a kind of cancer that occurred at the junction of distal esophagus and proximal stomach (1). In the past few decades, the incidence of GEJ cancer had remained high all over the world (2–4). Surgery treatment was the only way to cure GEJ cancer. Patients with early GEJ carcinoma could obtain a good prognosis after complete surgical resection of tumor tissue, while most advanced patients with Siewert II cancer who had infiltrated deep into the gastric wall, and developed lymph node metastasis (LNM) and distant metastasis had a poor prognosis (5–7). It was well known that surgery treatment could improve the prognosis of patients with GEJ cancer. Therefore, the purpose of this study was using the Surveillance, Epidemiology and End Results (SEER) database was to establish an effective prognostic nomogram to assess the prognosis of postoperative GEJ patients with no distant metastasis.

TNM staging system was first proposed by the Frenchman Pierre Denoix from 1943 to 1952, and now the American Joint Committee on Cancer (AJCC) and the Union for International Cancer Control (UICC) had gradually begun to establish international staging standards. In the eighth edition of AJCC TNM staging system, the TNM staging of breast cancer included the expression of estrogen receptor (ER) and progesterone receptor (PR), HER2 expression, tumor size, regional lymph node invasion and distant metastasis, which emphasized the role of clinical staging and pathological staging on prognosis (8). In the past two decades, the epidemiology of esophageal cancer and GEJ cancer had undergone profound changes, with a significant elevate in the incidence of adenocarcinoma and a gradual decrease in the incidence of squamous cell carcinoma. The two histological types were different in many characteristics including risk factors, tumor location, tumor biology and results (9). The SEER database was a publicly available, federally funded cancer reporting system. Compared with other common database, SEER data were national, population-based, and contain information on the clinicopathological characteristics of cancer cases (10). Zhou et al. discovered that the age, T stage, N stage, and examined lymph node were independent risk factors for poor prognosis in patients with GEJ adenocarcinoma without distant metastasis by the multivariate Cox regression for the clinical data of patients (N = 953) with GEJ adenocarcinoma in the SEER database from 1988 to 2011 (11). There were pathological types of squamous cell carcinoma in GEJ area. Therefore, we study retrospectively analyzed the clinical data of patients with GEJ cancer who had no distant metastasis and underwent surgery from 2010 to 2015 in the SEER database to predicte the risk factors affecting their overall survival (OS).

## Materials and Methods

### Patients and Study Design

The SEER database was an authoritative database of cancer incidence and prognosis information in the United States. In June 2020, we used SEER * Stat software to download the data of GEJ cancer patients based on the seventh edition of TNM staging from the SEER database, including diagnostic age range, sex, tumor grade, clinical stage, T stage, N stage, M stage, surgery or not, survival time and survival status. Then the analysis population was determined according to the following criteria: Surgery performed; Age range of diagnosis of GEJ cancer; Clear tumor grade, clinical stage, T stage and N stage. Patients with M1 stage GEJ cancer were excluded. 3425 GEJ cancer patients were segmented into the training and verification sets at 7:3 to construct and validate the nomogram.

### Construction and Identification of Nomogram

In the training cohort, univariate Cox regression was used to analyze the effects of the age, sex, tumor grade, clinical stage, T stage and N stage on the survival of patients with GEJ cancer without distant metastasis. On the basis of univariate Cox regression analysis, multivariate Cox regression analysis was applied to identify the independent risk factors related to survival in patients with GEJ cancer without distant metastasis. Survival analysis was used to identify the effects of the age, tumor grade, T stage and N stage on the prognosis of GEJ cancer patients without distant metastasis, and determined the valuable independent predictors of GEJ cancer patients without distant metastasis to construct a nomogram. Hazard ratios and 95% confidence intervals (CIs) were also calculated. Nomogram performance was quantified in terms of calibration and discrimination. The relationship between the actual probability and the predicted probability was evaluated *via* the calibration. The area under the curve (AUC) of the receiver operating characteristic (ROC) analysis and C index were used to evaluate the value of constructing the nomogram. The AUC value was between 0.5 and 1, and was positively correlated with prediction effect. In the process of external verification of the nomogram, we used multivariate Cox regression analysis to verify the results of the training cohort, and got the C index and calibration curve. Then the training and the verification cohorts were scored and divided into high- and low-risk groups *via* the Youden’s index (12). Kaplan-Meier survival analysis was applied to detect the effect of high- and low-risk groups on the prognosis of patients with GEJ cancer without distant metastasis.

### Statistical Analysis

The chi-square test was applied to evaluate the association between the training and the validation sets of GEJ cancer patients. A nomogram was built using R (version 3.6.1) package rms according to multivariate Cox regression analysis results. Kaplan-Meier survival analysis was applied to draw survival curves between the age tumor grade, T stage, N stage and risk score and the prognosis of patients with GEJ cancer without distant metastasis, and compared *via* the log-rank test. All results were visualized and analyzed using the GraphPad Prism 5.0 and R software. P < 0.05 was considered as statistically significant.

## Results

### Patient Baseline Characteristics

**Figure 1:**
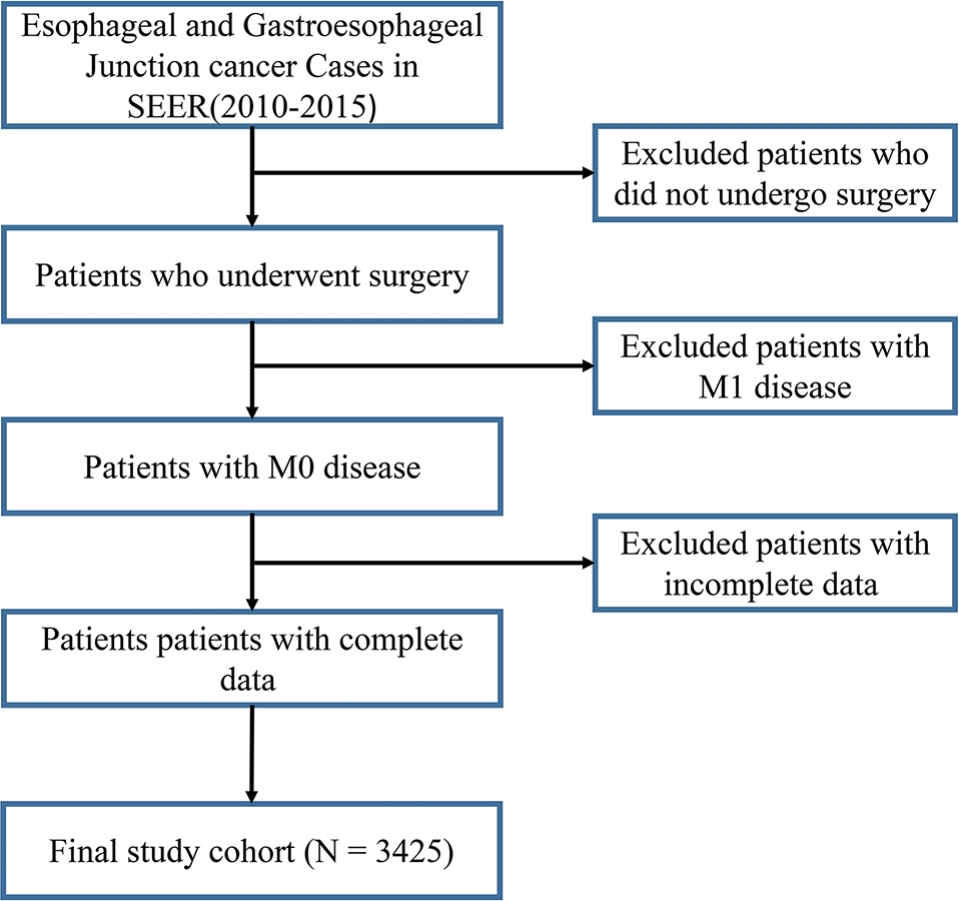
Selection of GEJ patients included in the study. GEJ, Gastroesophageal Junction, SEER, Surveillance, Epidemiology, and End Results.

**Figure 2:**
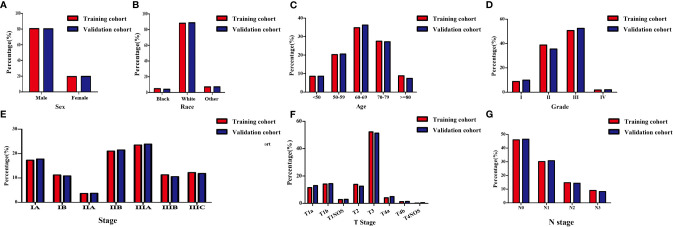
Demographic and clinicopathological characteristics of patients with gastroesophageal junction carcinoma without distant metastasis in the training and validation cohorts. **(A)** Sex; **(B)** Race; **(C)** Age; **(D)** Grade; **(E)** Clinical stage; **(F)** T stage; **(G)** N stage. T, depth of invasion; N, Lymph node metastasis.

In this study, 3425 patients with GEJ cancer diagnosed who underwent surgical resection without distant metastasis from 2010 to 2015 in the SEER database were enrolled according to screening criteria (**Figure 1**). The demographics and clinicopathologic characteristics were associated with the GEJ cancer included the age, race, sex, tumor grade, clinical stage, T stage, and N stage (**Table 1**). All patients were randomly assigned. 2400 patients were designed as training cohort and 1025 patients were designed as verification cohort. The characteristics of patients in each cohort were revealed in **Table 1**. The distribution proportion of the demographic and clinicopathological factors in the training cohort and the verification cohort tended to be the same (**Figure 2** and **Table 1**).

**Table 1:** Demographic and clinicopathological characteristics of patients with gastroesophageal junction carcinoma.

	Whole cohort	Training cohort	Validation cohort	P value
	N (%)	N (%)	N (%)	
All	3425 (100)	2400 (70.07)	1025 (29.93)	
Age				0.708
<50	293 (8.55)	205 (8.54)	88 (8.59)	
50–59	698 (20.38)	487 (20.29)	211 (20.59)	
60–69	1207 (35.24)	836 (34.83)	371 (36.20)	
70–79	939 (27.42)	660 (27.50)	279 (27.22)	
>=80	288 (8.41)	212 (8.83)	76 (7.41)	
Race				0.604
Black	159 (4.64)	117 (4.88)	42 (4.10)	
White	3024 (88.29)	2115 (88.13)	909 (88.68)	
Other	242 (7.07)	168 (7.00)	74 (7.22)	
Sex				0.941
Male	2756 (80.47)	1932 (80.50)	824 (80.39)	
Female	669 (19.53)	468 (19.50)	201 (19.61)	
Grade				0.294
I	313 (9.14)	211 (8.79)	102 (9.95)	
II	1294 (37.78)	930 (38.75)	364 (35.51)	
III	1753 (51.18)	1215 (50.63)	538 (52.49)	
IV	65 (1.90)	44 (1.83)	21 (2.05)	
Stage				0.991
IA	596 (17.40)	414 (17.25)	182 (17.76)	
IB	380 (11.09)	269 (11.21)	111 (10.83)	
IIA	125 (3.65)	87 (3.63)	38 (3.71)	
IIB	724 (21.14)	504 (21.00)	220 (21.46)	
IIIA	807 (23.56)	562 (23.42)	245 (23.90)	
IIIB	379 (11.07)	271 (11.29)	108 (10.54)	
IIIC	414 (12.09)	293 (12.21)	121 (11.80)	
Stage_T				0.617
T1a	409 (11.94)	276 (11.50)	133 (12.98)	
T1b	487 (14.22)	339 (14.13)	148 (14.44)	
T1NOS	97 (2.83)	67 (2.79)	30 (2.93)	
T2	462 (13.49	333 (13.88)	129 (12.59)	
T3	1779 (51.94)	1253 (52.21)	526 (51.32)	
T4a	138 (4.03)	98 (4.08)	40 (3.90)	
T4b	44 (1.28)	30 (1.25)	14 (1.37)	
T4NOS	9 (0.26)	4 (0.17)	5 (0.49)	
Stage_N				0.870
N0	1582 (46.19)	1105 (46.04)	477 (46.54)	
N1	1040 (30.36)	724 (30.17)	316 (30.83)	
N2	500 (14.60)	353 (14.71)	147 (14.34)	
N3	303 (8.847)	218 (9.08)	85 (8.29)	

N, The number of patients.

### Independent Predictors in the Training Cohort

Univariate Cox regression analysis revealed that the age, clinical stage, tumor grade, T stage and N stage were important risk factors affecting the prognosis of GEJ cancer patients without distant metastasis (**Figure S1** and **Table 2**). Multivariate Cox regression analysis displayed that the age, tumor grade, T stage and N stage were independent prognostic risk factors for patients with GEJ cancer without distant metastasis (**Figure S2** and **Table 3**).

**Table 2:** Univariate Cox regression analysis of the relationship between clinicopathological features and prognosis in gastroesophageal junction carcinoma patients.

Characteristic	HR	95% CI (Low)	95% CI (High)	P value
Age	1.156229113	1.094630521	1.221294068	2.03E-07
Race	0.9281553	0.829361079	1.038717975	0.194146632
Sex	1.092977802	0.938572749	1.272784103	0.252568539
Grade	1.430607678	1.307759489	1.564995968	5.40E-15
Stage	1.296696714	1.254525141	1.340285909	1.58E-53
Stage_T	1.337965946	1.278096466	1.400639875	1.15E-35
Stage_N	1.524187413	1.441596691	1.611509851	9.71E-50

HR, Hazard ratio; CI, Confidence interval.

**Table 3:** Multivariate Cox regression analysis of the relationship between clinicopathological features and prognosis in gastroesophageal junction carcinoma patients.

Characteristic	HR	95% CI (Low)	95% CI (High)	P value
Age	1.235389443	1.168671384	1.305916356	8.49E-14
Grade	1.156727967	1.048058445	1.276665054	0.003821989
Stage	1.064905211	0.941024138	1.2050946	0.31895049
Stage_T	1.169036138	1.059317348	1.290119051	0.001896703
Stage_N	1.248522934	1.078776224	1.444979489	0.002911148

HR, Hazard ratio; CI, Confidence interval.

**Figure 3:**
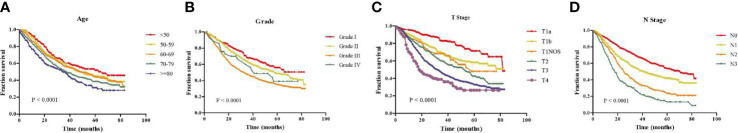
Kaplan-Meier survival analysis revealed that risk factors were concerned with the prognosis of patients with GEJ cancer without distant metastasis. **(A)** Age; **(B)** Tumor grade; **(C)** T stage; **(D)** N stage. GEJ, Gastroesophageal Junction; T, depth of invasion; N, Lymph node metastasis.

**Figure 4:**
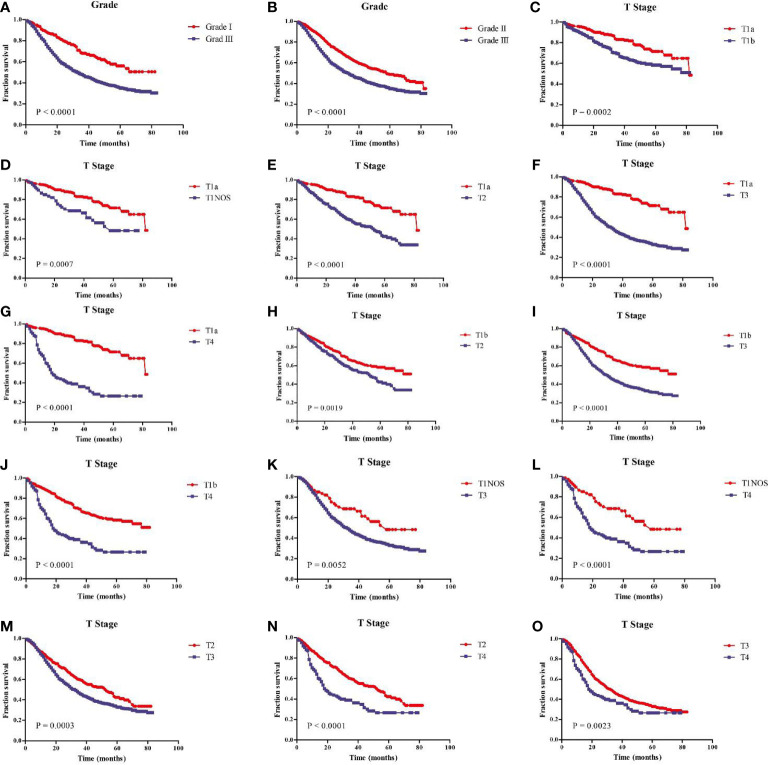
Kaplan-Meier survival analysis showed that prognostic risk factors tumor grade and T stage of patients with GEJ cancer without distant metastasis. **(A)** Grade I vs. III; **(B)** Grade II vs. III; **(C)** T1a vs. T1b; **(D)** T1a vs. T1NOS; **(E)** T1a vs. T2; **(F)** T1a vs. T3; **(G)** T1a vs. T4; **(H)** T1b vs. T2; **(I)** T1b vs. T3; **(J)** T1b vs. T4; **(K)** T1NOS vs. T3; **(L)** T1NOS vs. T4; **(M)** T2 vs. T3; **(N)** T2 vs. T4; **(O)** T3 vs. T4. GEJ, Gastroesophageal Junction; T, depth of invasion.

In addition, the age, tumor grade, T stage and N stage were respectively related to the OS of GEJ cancer patients without distant metastasis, and the difference was statistically significant (**Figure 3**). Subgroup analysis showed that compared with cancer patients aged <50, 50–59, and 60–69, patients with GEJ cancer without distant metastasis aged 70–79 and > = 80 had the worse prognosis, and the difference was statistically significant (**Figure S3**); Compared with Grade I and II cancer patients, Grade III patients with GEJ cancer without distant metastasis had the worse prognosis, and the difference was statistically significant (**Figures 4A, B**); compared with patients with stage T1a cancer, the prognosis of patients with GEJ cancer without distant metastasis in stage T1b, T1NOS, T2, T3, and T4 were worse (**Figures 4C–G**). Compared with patients with stage T1b cancer, patients with stage T2, T3, and T4 GEJ cancers without distant metastasis had the worse prognosis (**Figures 4H–J**). Compared with patients with stage T1NOS cancer, patients with stage T3 and T4 GEJ cancer without distant metastasis had the worse prognosis (**Figures 4K, L**). Compared with patients with stage T2 cancer, patients with stage T3 and T4 GEJ cancer without distant metastasis had the worse prognosis (**Figures 4M, N**). Compared with patients with stage T3 cancer, patients with stage T4 GEJ cancer without distant metastasis had the worse prognosis, and the difference was statistically significant; compared with patients with stage N0 cancer, the prognosis of patients with stage N1, N2, and N3 GEJ cancer without distant metastasis was worse (**Figures S4A–C**). Compared with patients with stage N1 cancer, patients with stage N2 and N3 GEJ cancer without distant metastasis had the worse prognosis (**Figures S4D–F**). Compared with patients with stage N2 cancer, patients with stage N3 GEJ cancer without distant metastasis had the worse prognosis, and the difference was statistically significant (**Figure S4F**).

### Construction of Predictive OS Model

**Figure 5:**
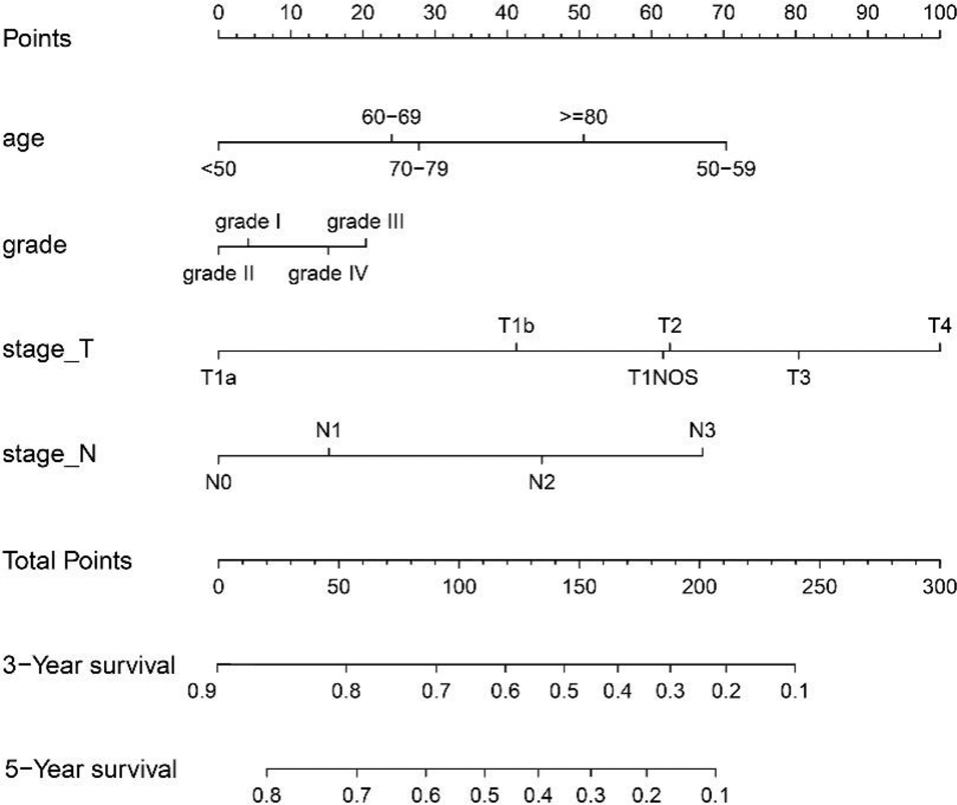
Construction of nomogram in the training cohort.

**Figure 6:**
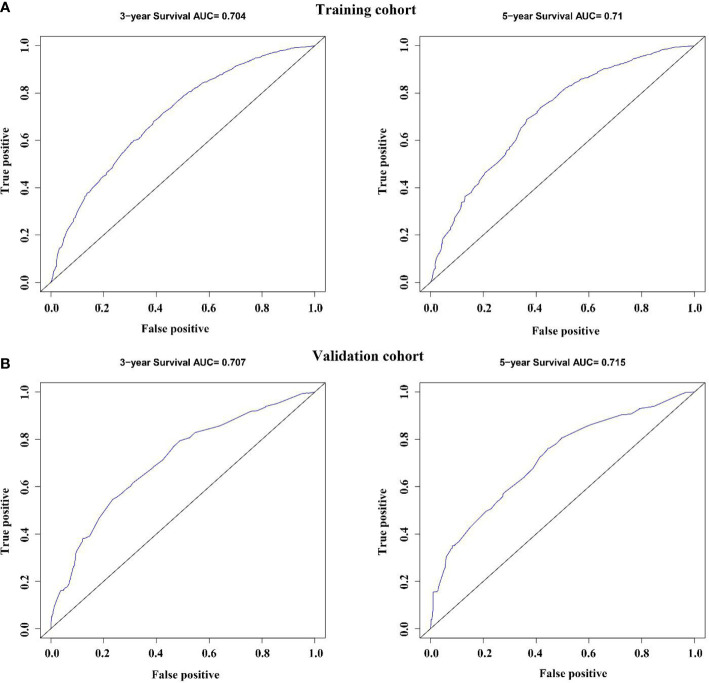
Validation of the nomogram *via* the receiver operating characteristic curves. **(A)** Training cohort; **(B)** Validation cohort.

**Figure 7:**
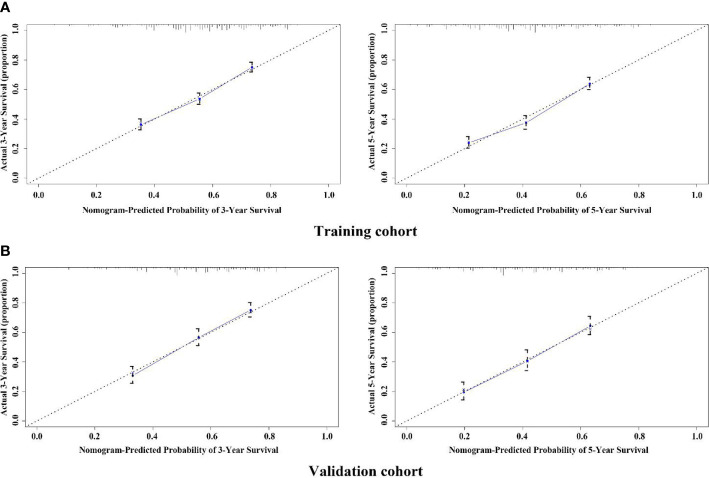
Calibration plot of the nomogram for assessing the survival of GEJ cancer without distant metastasis in the training and validation cohorts. **(A)** Training cohort; **(B)** Validation cohort.

The variables with statistical differences in multivariate Cox regression analysis were incorporated into the nomogram model, and the nomogram model was constructed by integrating the independent predictors, including the age, grade, T stage and N stage (**Figure 5**). The results revealed that T stage had the greatest contribution to the prognosis of GEJ cancer patients without distant metastasis. A score was given on the point scale axis, and the total score of the individual patient could be easily calculated by adding each score. Therefore, it was possible to estimate the 3- and 5-year OS probabilities per patient with GEJ cancer without distant metastasis. The C index of nomogram model was 0.667. The AUC of 3- and 5-year OS were 0.704 and 0.71, respectively (**Figure 6A**). The calibration curve of postoperative 3- and 5-year OS revealed the best consistency between nomogram predictions and actual observations (**Figure 7A**).

### Verification of the Accuracy of Nomogram in Predicting OS

In the validation cohort, the age, sex, race, grade, tumor stage, T stage and N stage of GEJ patients without distant metastasis tended to be consistent with the proportion of the training cohort (**Figure 2** and **Table 1**). The verification cohort predicted that the nomogram C index of OS was 0.663. The AUC of 3- and 5-year OS were 0.707 and 0.715, respectively (**Figure 6B**). The calibration curve of 3- and 5-year OS showed the best consistency between Nomogram predictions and actual observations, and the verification cohort was consistent with the training cohort (**Figure 7B**).

### Risk Stratification *via* the Nomogram

**Figure 8:**
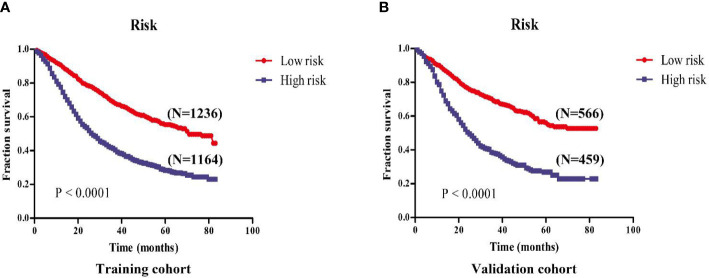
Kaplan-Meier survival analysis revealed that the prognosis of patients in the high- and low-risk groups in the training and validation cohorts. **(A)** Training cohort; **(B)** Validation cohort.

The cut-off value of total scores for predicting GEJ cancer without distant metastasis was decided *via* the Youden’s index in the training cohort. Both the training and validation cohorts were segmented into the low- and high-risk groups. There was a significant difference in the OS of GEJ cancer without distant metastasis between the low- and high-risk groups in the training and validation cohorts (**Figure 8**).

## Discussion

AJCC staging system is often used to assess the prognosis and clinical treatment effect of patients with esophageal cancer, lung cancer, liver cancer and so on (8, 9, 13–15). However, tumor patients will have different survival times because of individual heterogeneity, so it is inaccurate to only rely on AJCC staging system to predict survival time of tumor patients. At present, the AJCC staging system does not take into account the age, sex, race and so on of cancer patients, but multicenter data showed that the age, sex and race were closely relevant to the prognosis of cancer patients (16–19). Previous studies had reported that prognostic models for patients with esophageal cancer and type II of GEJ adenocarcinoma had been constructed, but there was a lack of prognostic models for postoperative patients with GEJ cancer without distant metastasis (20–22). Therefore, we screened the clinicopathological characteristic data of patients with GEJ cancer in SEER database through inclusion and exclusion criteria, and established nomogram to assess the OS of patients with GEJ cancer, in order to develop a richer and more accurate prognostic model to evaluate the survival and clinical treatment of patients with GEJ cancer.

A total of 3425 surgically treated GEJ cancer patients without distant metastasis were enrolled in this study. Through random sampling, 70% of the patients (N = 2400) were designed as training cohort, and the rest of the patients were designed as verification cohort. The age, tumor grade, T stage and N stage were identified as independent prognostic risk factors by univariate and multivariate Cox regression analysis. In this study, the age, tumor grade, T stage and N stage were significantly correlated with the OS in patients with GEJ cancer, while gender, race and clinical stage were not significantly correlated with the OS in patients with GEJ cancer. The possible reasons were the difference of sample size and the selection deviation of included variables.

According to the multivariate Cox regression analysis results, significant variables were selected to construct nomogram for indicating the OS of tumor patients. The results displayed that the age, tumor grade, T stage and N stage were significantly connected with OS in patients with GEJ carcinoma. As shown in the model, T stage had the greatest impact on the prognosis of patients with GEJ carcinoma. It is undeniable that the AJCC staging system is still the most important tool for predicting patient survival and guiding the treatment of cancer patients, but it does not fully consider the impact of demographic information and clinicopathological features on patient prognosis. In this study, the age was an independent prognostic risk factor for postoperative GEJ cancer patients without distant metastasis. At the same time, nomogram model suggested that N stage was an important prognostic factor in patients with GEJ cancer, and it was suggested that patients with GEJ cancer should undergo regional lymph node examination regularly. In order to ensure the versatility of the prediction model, we used C index to evaluate the effectiveness of the model. By comparing the predicted probability map of nomogram with the actual probability map, the effectiveness of the model was evaluated. In this study, the C index of OS predicted by nomogram model was 0.667, which was almost the same as the C index 0.697 of the seventh edition AJCC staging system for esophageal cancer (23). In addition, Feng et al. reported that the C index of type II GEJ adenocarcinoma prognostic model was 0.55 and the eighth edition TNM staging C index was 0.61 (22), which indicated that the predictive model had more accurate predictive value. The results based on the verification cohort showed that the 3- and 5-year OS probability predictions were in good agreement with the observed results, which ensured that the constructed Nomogram had high reliability and repeatability.

This study also had some shortcomings. First of all, as a retrospective analysis, there were some limitations in data acquisition. Secondly, there was variable selection bias in research and design. There was no molecular information of HER2/Neu overexpression in SEER database. It had been proved to be related to the prognosis of patients with esophageal carcinoma (24). At the same time, the SEER database did not contain complete treatment records in our research, such as chemotherapy options or targeted treatment information. In addition, some factors tested in the laboratory, such as tumor markers, were also important for affecting the survival time of cancer patients. However, our study has certain advantages. Compared with the study by Zhou et al., our study had a large sample. Zhou et al. found that age, depth of invasion, the number of metastasized lymph nodes, and the number of examined lymph nodes were the independent prognostic factors, and sex and tumor grade were not prognostic-related risk factors and not statistically significant. However, their constructed nomogram containd sex and tumor grade of cancer patients (11). Our nomogram model was built on the basis of univariate and multivariate Cox regression analysis, with stricter screening. The nomogram proposed in this study could objectively and accurately assess the prognosis of patients with GEJ carcinoma without distant metastasis. The model was based on easily accessible variables, was convenient to use, and had better ability of prognosis identification and survival prediction. it could be used to calculate individual tumor-specific survival prediction and provide better treatment allocation for patients.

## Data Availability Statement

Publicly available data was analyzed, and the data generated can be accessed in the SEER database (https://seer.cancer.gov/) for this study.

## Author Contributions

JLG designed the experiment and explained the data. QG processed and analyzed the SEER data and wrote the manuscript. JLG and YYP guided the revision and language modification of the manuscript. HY processed and edited pictures. All authors contributed to the article and approved the submitted version.

## Funding

This work was subsidized by the scientific and technological research and development project plan of Shiyan city, Hubei Province (no. 16Y10).

## Conflict of Interest

The authors declared that the research was conducted in the absence of any commercial or financial relationships that could be construed as a potential conflict of interest.
