# Supplementary material for: Prognostic Nomogram for Postoperative Patients With Gastroesophageal Junction Cancer of No Distant Metastasis
Source: Front Oncol. 2021 Apr 16;11:643261. doi: 10.3389/fonc.2021.643261 (PMC8085428; doi:10.3389/fonc.2021.643261)
Supplement: Supplementary file 1 [file DataSheet_1.docx]

Supplementary Material

**
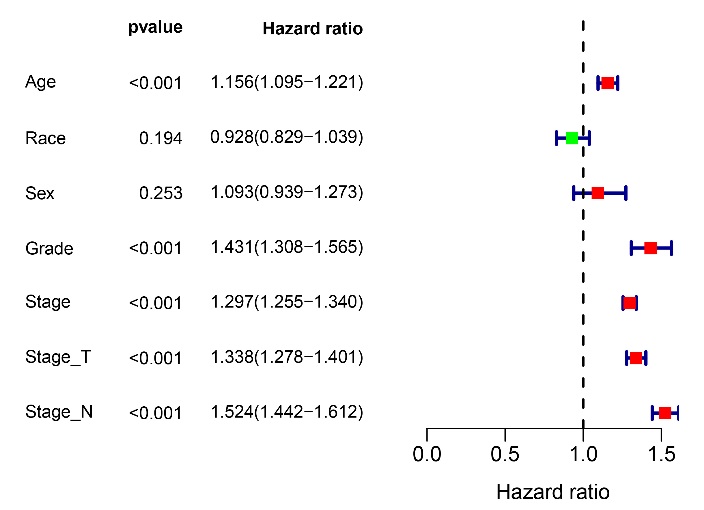
**

Figure S1. The risk factors affecting the prognosis of patients with GEJ cancer without distant metastasis via univariate Cox regression analysis. GEJ, Gastroesophageal Junction. T stands for depth of invasion; N, Lymph node metastasis.


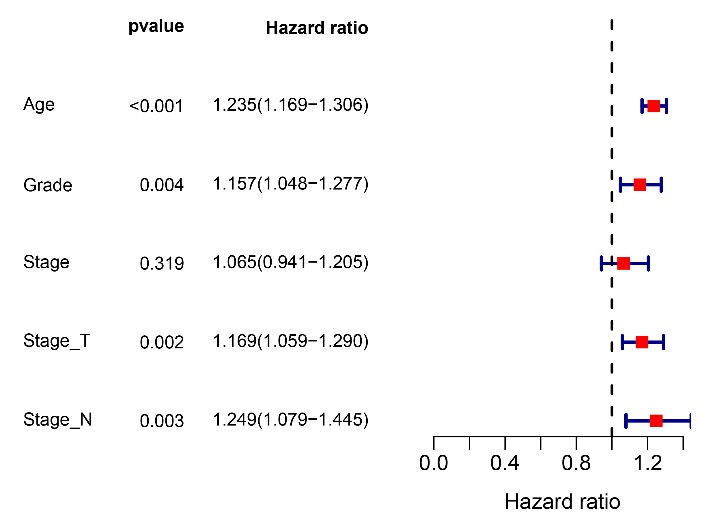


Figure S2. The risk factors affecting the prognosis of patients with GEJ cancer without distant metastasis via multivariate Cox regression analysis. GEJ, Gastroesophageal Junction. T stands for depth of invasion; N, Lymph node metastasis.


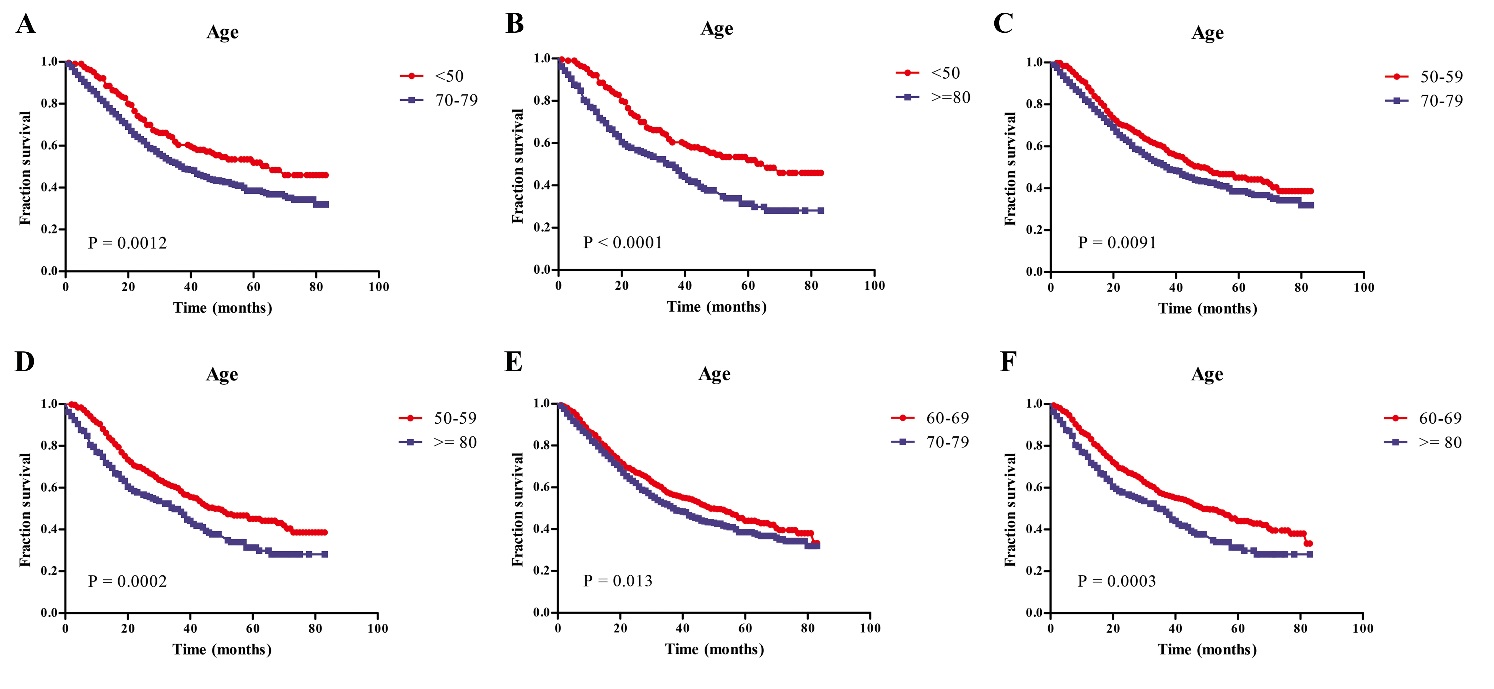


Figure S3. Kaplan-Meier survival analysis showed that prognostic risk factors age of patients with GEJ cancer without distant metastasis. (A) < 50 vs 70-79; (B) < 50 vs >=80; (C) 50-59 vs 70-79; (D) 50-59 vs >=80; (E) 60-69 vs 70-79; (F) 60-69 vs >=80. GEJ, Gastroesophageal Junction.


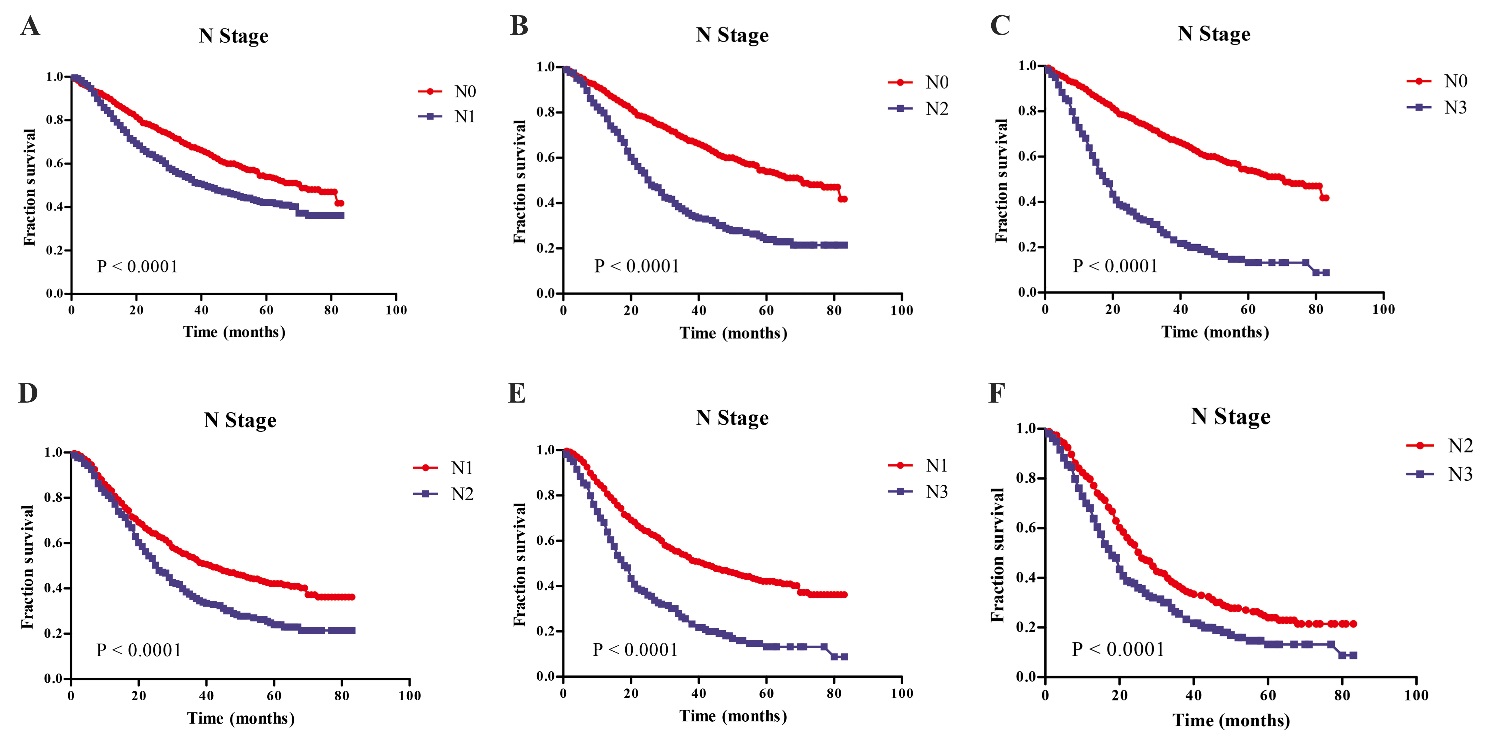


Figure S4. Kaplan-Meier survival analysis showed that prognostic risk factors N stage of patients with GEJ cancer without distant metastasis. (A) N0 vs N1; (B) N0 vs N2; (C) N0 vs N3; (D) N1 vs N2; (E) N1 vs N3; (F) N2 vs N3. GEJ, Gastroesophageal Junction; N stands for lymph node metastasis.
